# Combination of Colchicine and Ticagrelor Inhibits Carrageenan-Induced Thrombi in Mice

**DOI:** 10.1155/2022/3087198

**Published:** 2022-01-17

**Authors:** BuChun Zhang, Rong Huang, DaiGang Yang, GuiLan Chen, YuanLi Chen, Jihong Han, Shuang Zhang, LiKun Ma, XiaoXiao Yang

**Affiliations:** ^1^Department of Cardiology, The First Affiliated Hospital of USTC, Division of Life Sciences and Medicine, University of Science and Technology of China, Hefei, Anhui, China; ^2^Key Laboratory of Metabolism and Regulation for Major Diseases of Anhui Higher Education Institutes, College of Food and Biological Engineering, Hefei University of Technology, Hefei, China

## Abstract

The formation of a thrombus is closely related to oxidative stress and inflammation. Colchicine is one of the most commonly prescribed medication for gout treatment, with anti-inflammation and antioxidative stress properties. Therefore, we speculated that it is possible for colchicine to treat thrombosis. In this study, we used carrageenan to induce thrombosis in BALB/c mice and fed mice with colchicine, ticagrelor, and their combination, respectively. We found colchicine inhibited carrageenan-induced thrombi in mouse tail, and the inhibition was enhanced by ticagrelor. *In vitro*, colchicine inhibited thrombin-induced retraction of human platelet clots. Mechanically, colchicine inhibited platelet activation by reducing the expression of platelet receptors, protease-activated receptor 4 (PAR4) and CD36, and inactivating of AKT and ERK1/2 pathways. Furthermore, in human umbilical vein endothelial cells (HUVECs), colchicine showed antioxidative stress effects through increasing protein expression of glutathione peroxidase-1 (GPx-1), and mRNA levels of forkhead box O3 (FOXO3a) and superoxide dismutase 2 (SOD2). In RAW264.7 cells, colchicine reduced LPS-enhanced inflammatory response through attenuating toll-like receptor 4 (TLR4) activation. In addition, colchicine reduced LPS or ox-LDL-induced monocyte adhesion to HUVECs by inhibiting intercellular adhesion molecule-1 (ICAM-1) and vascular adhesion molecule-1 (VCAM-1) levels. Taken together, our study demonstrates that colchicine exerts antithrombotic function by attenuating platelet activation and inhibiting oxidative stress and inflammation. We also provide a potential new strategy for clinical treatment.

## 1. Introduction

A thrombus is a blood clot that forms in the vessels due to coagulation disorder. It can cause tissue infarction and other serious complications, such as cerebral embolism and pulmonary embolism, which seriously affect human health. In the past decades, aspirin, clopidogrel, prasugrel, and ticagrelor have been widely used worldwide for cardiovascular diseases due to their effective antiplatelet aggregation effects [1]. Current clinical studies suggest that ticagrelor combined with aspirin is superior to clopidogrel in the prevention of acute coronary syndrome [2]. However, these drugs may cause severe side effects including gastrointestinal bleeding, dyspnea, and gout. Therefore, new or better solutions are still needed for antithrombotic therapy.

The process of thrombosis is complicated. Platelet activity is associated with initiation of the coagulation cascade, which can be activated in response to vascular damage. In pathophysiological states, platelets are overactivated to produce soluble agonists, such as adenosine diphosphate (ADP), thromboxane A2, and thrombin, which can further promote platelets adhere to subendothelial exposed collagen, thereby mediating excessive aggregation to form thrombi. Thrombin is essential in thrombosis mainly through activation of protease-activated receptors (PARs). In addition, other platelet surface receptors, such as CD36, CD41, CD42, and CD61, can also promote the development of a thrombus [3].

General risk factors of thrombosis include hypertension, high levels of low-density lipoprotein- (LDL-) cholesterol, and smoking. At the same time, diabetes, pregnancy, obesity, age, chemotherapeutics, infectious burden, and human immunodeficiency virus also increase the risk [4]. In addition, oxidative stress and inflammation are also major players in platelet activation and subsequent thrombosis [5]. Abnormal elevation of reactive oxygen species (ROS) is associated with oxidative stress [6]. In the process of vascular injury, ROS can be produced by vascular cells, such as endothelial cells, smooth muscle cells, and fibroblasts, to stimulate platelet activation. Furthermore, activated platelets, monocytes, and endothelial cells secrete adhesion molecules, such as vascular cell adhesion molecule-1 (VCAM-1) and intercellular adhesion molecule-1 (ICAM-1), which can trigger platelet or monocyte adhesion to endotheliocytes for accelerating the aggregation of platelets to injured endotheliocytes. ROS accumulation is also accompanied by inflammation, which can promote the secretion of inflammatory cytokines. Toll-like receptor 4 (TLR4) plays a key role in inflammatory response. In RAW264.7 cells, fibrinogen stimulates the expression of macrophage inflammatory protein-1a (MIP-1*α*), MIP-1*β*, MIP-2, and monocyte chemotactic protein-1 (MCP-1) *via* TLR4 [7]. It has been shown that a-ketoglutarate inhibits thrombosis by reducing serum inflammatory cytokines IL-1b, IL-6, TNF-*α*, and leukocyte accumulation in lung tissue [8]. Moreover, thrombin cleaves fibrinogen to produce fibrin and activates PARs that can further contribute to inflammatory response in the process of thrombus. AKT and ERK1/2 pathways are critical for many cellular processes, including cell survival, oxidative stress, metabolism, and migration [9]. A previous study has shown that AKT1 knockout mice displayed an increased bleeding time and inhibited agonist-induced platelet aggregation *in vitro* [10]. In addition, dihydromyricetin leads to impaired endothelial activation and defective platelet activation by inhibiting agonist-induced phosphorylation of ERK1/2 and p38 [11].

Colchicine is a classical anti-inflammation drug extracted from the autumn crocus and has been widely used in the treatment of gout [12]. Clinical trials have demonstrated that colchicine can inhibit atherosclerotic plaque rupture by reducing neutrophil aggregation [13]. It has been reported that colchicine improves renal ischemia-reperfusion injury-induced liver dysfunction by regulating hepatic ROS metabolism [14]. In addition, a recent study indicates that colchicine can inhibit platelet-rich plasma ROS generation in response to collagen glycoprotein VI stimulation [15]. In this study, we used carrageenan to induce thrombosis in mice. Considering the strong antiplatelet effect and long half-life of ticagrelor, we chose it as the positive control drug in this study. To reveal if colchicine can inhibit or prevent carrageenan-induced thrombosis, we treated mice with colchicine before or after carrageenan injection. In addition, we also attempt to determine if the cotreatment of colchicine and ticagrelor has better preventive and protective effects on thrombosis.

## 2. Materials and Methods

### 2.1. Reagents

Ticagrelor was purchased from AstraZeneca (London, UK). Colchicine and prostaglandin E1 (PGE1) were purchased from MedChemExpress (NJ, USA). Buthionine sulfoximine (BSO) was purchased from Cayman Chemical (Ann Arbor, MI). Fibrinogen and thrombin were purchased from Sigma-Aldrich (St. Louis, MO, USA). LY294002 was purchased from Selleck (Boston, MA, USA). U0126 was purchased from LC Laboratories (Woburn, MA, USA). ROS assay kit was purchased from Beyotime (Nantong, China). 5,6-Carboxyfluorescein diacetate succinimidyl ester (CFSE) was purchased from Santa Cruz Biotechnology (Dallas, TX, USA). 4′,6-Diamidino-2-phenylindole (DAPI) was purchased from Santa Cruz Biotechnology (Paso Robles, CA, USA).

Rabbit anti-AKT (Cat# 10176-2-AP, 1 : 5000), phosphorylated AKT (p-AKT, Ser473; Cat# 66444-1-lG, 1 : 5000), PAR4 (Cat# 25306-1-AP, 1 : 2000), and catalase (CAT, Cat# 19792-1-AP, 1 : 2000) polyclonal antibodies were purchased from Proteintech Group (Chicago, IL, USA). Rabbit anti-ERK1/2 (Cat# 9102S, 1 : 1000) and phosphorylated ERK1/2 (p-ERK1/2, Thr202/Tyr204; Cat# 9101S, 1 : 1000) polyclonal antibodies were purchased from Cell Signaling Technology (Danvers, MA, USA). Rabbit anti-CD36 (Cat# NB400-145, 1 : 2000) monoclonal antibody was purchased from Novus (St Louis, MO, USA). Rabbit anti-CD41 (Cat# A5680, 1 : 2000), glutathione peroxidase 1 (GPx-1, Cat# A1110, 1 : 2000), superoxide dismutase 2 (SOD2, Cat# A1340, 1 : 2000), interleukin-1*β* (IL-1*β*, Cat# A17361, 1 : 2000) polyclonal, and mouse anti-glyceraldehyde-3-phosphate dehydrogenase (GAPDH, Cat# AC033, 1 : 100000) monoclonal antibodies were purchased from ABclonal (Wuhan, China). Goat anti-rabbit IgG- (whole molecule-) FITC (Cat# F0382) was purchased from Sigma-Aldrich. Rhodamine phalloidin was purchased from Yeason (Shanghai, China).

### 2.2. Cell Culture

HUVECs, THP-1 monocytes (a human monocytic cell line), and RAW264.7 cells were purchased from ATCC (Manassas, VA, USA). HUVECs were cultured in DMEM medium, THP-1, and RAW264.7 cells in RPMI 1640 medium, which contained 10% fetal bovine serum, 50 *μ*g/mL penicillin, and 50 *μ*g/mL streptomycin. Cells at 90% confluence were switched to serum-free medium before indicated treatment.

### 2.3. Induction of Thrombosis in Mice by Carrageenan Injection

BALB/c mice (male, ~7 weeks old, ~25 g) were purchased from GemPharmatech LLC (Nanjing, China). The protocols for animal studies (#HFUT20210403001) were approved by the Institution Animal Ethics Committee of Hefei University of Technology (Hefei, China). The studies were performed in compliance with the Guide for the Care and Use of Laboratory Animals published by the NIH.

**Figure 1:**
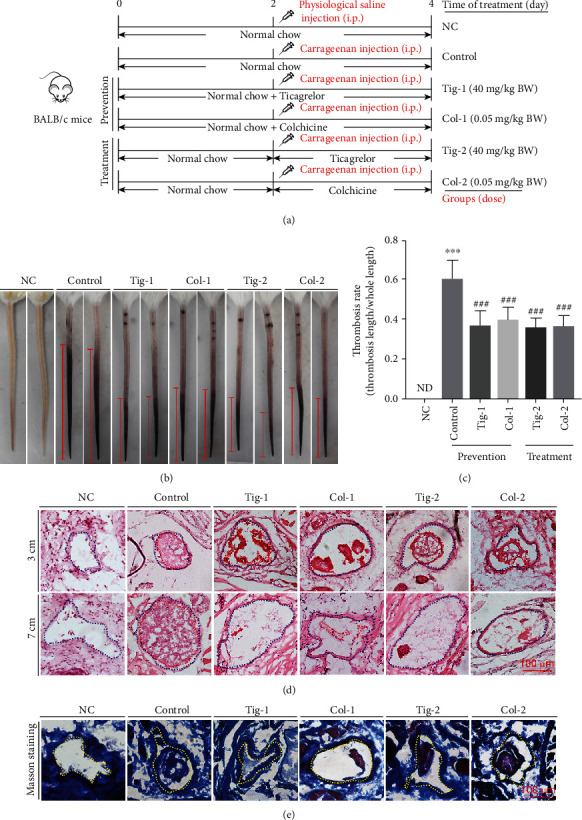
Colchicine inhibits carrageenan-induced thrombosis in mouse tail. (a) Experimental design: BALB/c mice in 6 groups (6/group) received the following treatment; negative control group (NC) and control group (Control): i.g. administration of PBS; Tig-1 and Col-1 (prevention groups): i.g. administration of ticagrelor (40 mg/day/kg bodyweight, Tig-1 group) or colchicine (0.05 mg/day/kg bodyweight, Col-1 group) for 4 days; Tig-2 and Col-2 (treatment groups): i.g. administration of ticagrelor (Tig-2 group) or colchicine (Col-2 group) for the last 2 days of treatment. Mice were i.p. injected with carrageenan solution (50 mg/kg bodyweight) on day 2, except for the NC group. Two days after carrageenan injection, mouse tail samples were collected. (b, c) The tail of each mouse was photographed, and the representative pictures were shown (b); the thrombosis rate (the ratio of tail length with thrombus to whole tail length) was calculated (c). (d, e) the cross sections of tail at different tail locations were conducted HE staining (d) and at the location of 3 cm were conducted Masson staining (e). The blue dotted line represents the outline of the blood vessel, and the red dotted line represents the intravenous thrombus. ND: not detected; ^∗∗∗^*P* < 0.001 vs. NC group; ^###^*P* < 0.001 vs. control group (*n* = 6).

To determine the prevention and treatment effects of colchicine and ticagrelor in blood vessels of mouse tail, we randomly divided BALB/c mice into 6 groups (6 mice/group) and treated them with ticagrelor (40 mg/kg bodyweight/day) or colchicine (0.05 mg/kg bodyweight/day), respectively. Dosage of colchicine and ticagrelor corresponded to the usage in human beings and previous studies [16–18]. Mice have received the treatment as indicated in Figure 1(a). Briefly, mice in the negative control group (NC) and the control group (control) received i.g. administration of PBS for 4 days; prevention groups (Tig-1 and Col-1) received i.g. administration of ticagrelor and colchicine for 4 days, respectively; treatment groups (Tig-2 and Col-2) received i.g. administration of ticagrelor and colchicine for 2 days, respectively. Mice in the NC group were i.p. injected with PBS, others were i.p. injected with carrageenan solution (50 mg/kg bodyweight) on day 2 to construct a thrombosis mouse model. At the end of experiment, all mice were anesthetized in a CO_2_ chamber followed by photograph of tail and collection of tail samples.

**Figure 2:**
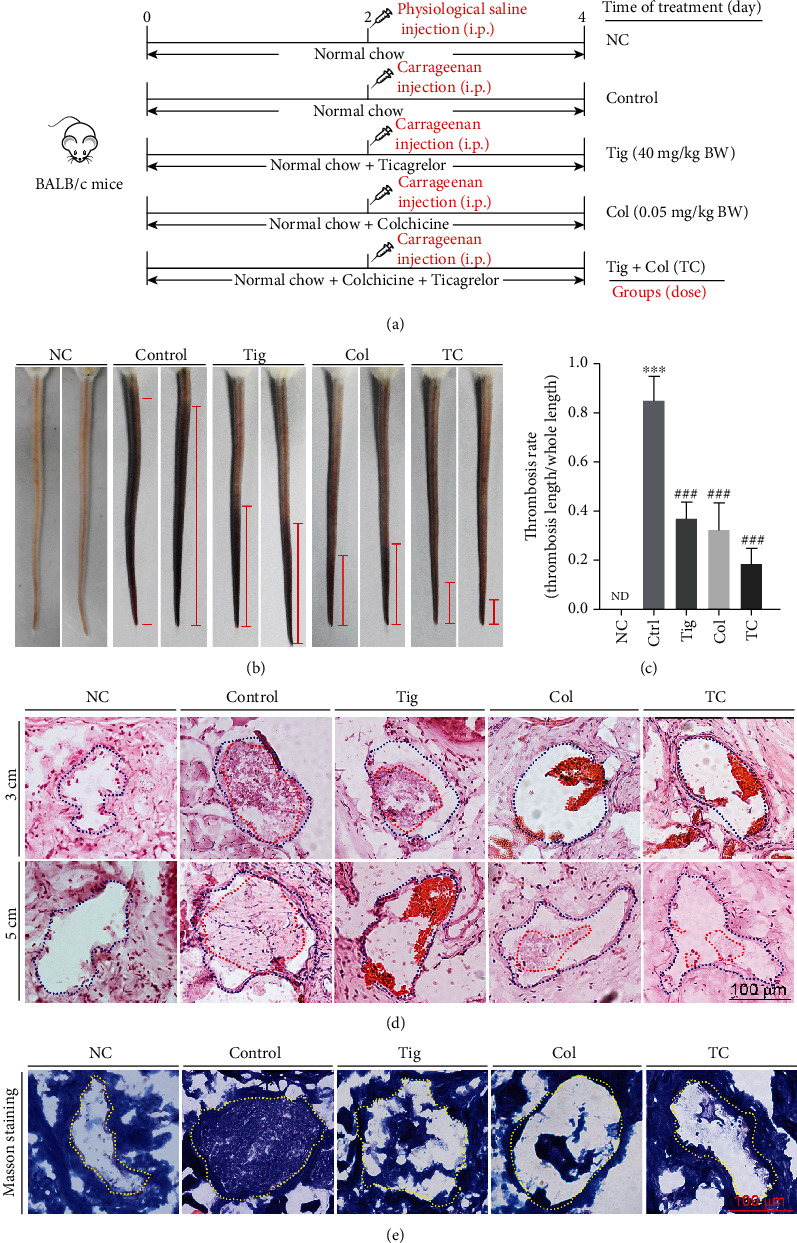
The combination of ticagrelor and colchicine inhibits carrageenan-induced thrombosis in mouse tail. (a) Experimental design: BALB/c mice in 5 groups (5/group) received the following treatment; negative control group (NC) and control group (Control): i.g. administration of PBS; Tig group: i.g. administration of ticagrelor (40 mg/day/kg bodyweight) for 4 days; Col group: i.g. administration of colchicine (0.05 mg/day/kg bodyweight) for 4 days; TC group: i.g. administration of ticagrelor and colchicine for 4 days. Mice were i.p. injected with carrageenan solution (50 mg/kg bodyweight) on day 2, except for those in the NC group. Two days after carrageenan injection, all mice were sacrificed followed by collection of blood, tail, liver, and lung samples individually; (b, c) tail from each mouse was photographed, and the representative pictures were presented (b). The thrombosis rate was calculated (c). (d, e) The cross sections of tail at different tail locations were conducted HE staining (d) and at the location of 3 cm were conducted Masson staining (e). ND: not detected; ^∗∗∗^*P* < 0.001 vs. NC group; ^###^*P* < 0.001 vs. control group (*n* = 5).

To determine combination treatment effects of colchicine and ticagrelor in blood vessels of mouse tail, liver, and lung tissues, we randomly divided BALB/c mice into 5 groups (5 mice/group). Mice were treated with PBS, colchicine, ticagrelor, and the combination of colchicine and ticagrelor (TC) for 4 days as indicated in Figure 2(a). After 48 h of PBS or carrageenan injection, all mice were anesthetized in a CO_2_ chamber, then the images of mouse tail were photographed, and tail, blood, liver, and lung samples were collected.

### 2.4. Detection of Liver Function Indexes

Serum was collected from the blood for determination of alanine aminotransferase (ALT) and aspartate aminotransferase (AST) levels using an automatic biochemical analyzer (Model 7020, Hitachi, Tokyo, Japan).

### 2.5. Hematoxylin and Eosin (HE) and Masson Staining

At the end of experiment, a piece of mouse tail at the indicated locations (the distance from the tail tip), liver or lung tissue was collected and fixed in 4% paraformaldehyde overnight. Samples were incubated in a 30% sucrose solution overnight and then embedded in OCT solution. To determine thrombi formed within mouse tissue vessels, the 5 *μ*m frozen tissue sections were conducted HE staining [19]. To determine the collagen content, the 5 *μ*m frozen mouse tail sections were conducted Masson staining with the corresponding assay kit (Solarbio, Beijing, China). The images of HE and Masson staining were photographed with a Zeiss microscope (Oberkochen, Germany).

### 2.6. Isolation of Platelet

The protocol for study with human platelets was prepared in accordance with the Code of Ethics of the World Medical Association and approved by the Clinical Ethics Committee of the First Affiliated Hospital of USTC (Hefei, Anhui, China). Human plasma was obtained from a healthy donor (The First Affiliated Hospital of USTC, Hefei, Anhui, China). Platelet was collected from plasma as described [20].

C57BL/6J mouse blood was used to obtain murine platelets. Mice in 2 groups (6 mice/group) received i.g. administration of 150 *μ*L PBS or colchicine solution (0.05 mg/day/kg bodyweight) for 2 days. After treatment, blood was collected for preparation of platelet [20].

### 2.7. Determination of Monocyte Adhesion to HUVECs

THP-1 monocytes (1 × 10^5^ cells/well) were labeled with CFSE (5 *μ*M) for 30 min. HUVECs cultured in 24-well plates were pretreated with the indicated drugs or their combination overnight, then cocultured with labeled THP-1 cells for 1 h at 37°C, followed by washing with PBS for 3 times. The adherent monocytes to HUVECs were observed under a Leica microscope (Wetzlar, German) and photographed.

### 2.8. Determination of ROS Levels in HUVECs and RAW264.7 Cells

After treatment, HUVECs or RAW264.7 cells in 24 or 96 plates were incubated with DCFH-DA solution (5 *μ*M) for 20 min in the dark, then washed with PBS for 3 times. Cells in 96 plates were used to detect cellular ROS levels by measuring of fluorescence intensity with a fluorescence microplate reader (EnSpire, PerkinElmer Life Sciences, USA); those in 24 plates were photographed with a Zeiss microscope (Oberkochen, Germany) [21].

### 2.9. Clot Retraction Assay

Human platelets (3 × 10^8^/mL) were suspended in buffer solution (10 mM HEPES, pH 7.4, 140 mM NaCl, 3 mM KCl, 0.5 mM MgCl_2_, 5 mM NaHCO_3_, and 10 mM glucose) at 37°C, then added with CaCl_2_ (1 mM), followed by PBS or colchicine (100 ng/mL) treatment at 37°C for 30 min. After treatment, fibrinogen (2 mg/mL) was added to the suspension thoroughly mixed, followed by adding thrombin (1 U/mL) to trigger clot retraction. Clots were photographed at the different time points.

### 2.10. Immunofluorescent Staining

The 96-well plate covered with slides was precoated with fibrinogen (20 *μ*g/mL) at 4°C overnight. Mouse platelets were transferred to the plate and incubated at 37°C for 1 h, then washed with PBS. The adherent platelets were conducted with immunofluorescent staining by anti-CD41 or CD36 antibody. The slides were mounted, and images were photographed with a Zeiss microscope (Oberkochen, Germany).

### 2.11. Determination of Protein or mRNA Expression by Western Blot or Quantitative Real-Time PCR (qRT-PCR)

After treatment, human platelets, HUVECs, or RAW264.7 cells were lysed with lysis buffer. Protein concentration was determined by the BCA protein assay kit. The same amount of protein from each sample was used to determine PAR4, CD36, GPx-1, CAT, SOD2, TLR4, IL-1*β*, p-ERK1/2, ERK1/2, p-AKT, AKT, and GAPDH protein expression by Western blot as described [22]. The signals were detected by Chemiscope 3000 mini (Qinxiang, Shanghai, China), and band density was quantified by Photoshop software.

After treatment, total RNA was extracted from HUVECs or RAW264.7 cells using Trizol. cDNA was synthesized with the same amount of total RNA from each sample by HiScript II Q Select RT SuperMix (+gDNA wiper). qRT-PCR was performed using the AceQ SYBR qPCR Master Mix on LightCycler96 (Roche, Mannheim, Baden-Württemberg, Germany) with indicated primers listed in Table 1. mRNA expression was normalized by GAPDH mRNA in the corresponding samples.

### 2.12. Data Analysis

All the data were generated from at least 3 independent experiments. Data were presented as mean ± S.E.M. GraphPad Prism 7.0 was used for statistical analysis. All the data in the normal distribution were analyzed by unpaired Student's *t*-test after evaluation of variance homogeneity. The significant difference was considered if *P* < 0.05.

## 3. Results

### 3.1. Colchicine Inhibits Carrageenan-Induced Thrombosis in Mouse Tail

To determine the preventive and therapeutic effects of colchicine on thrombosis, BALB/c mice were scheduled the treatment indicated in Figure 1(a). After 2 days of carrageenan injection, the mouse tail was photographed. Compare to the NC group mice, carrageenan induced a severe thrombosis formation in the mouse tail of the control group (Figures 1(b) and 1(c)). In contrast, the length of tail with thrombosis was much shorter in mice receiving ticagrelor or colchicine pretreatment (Tig-1 and Col-1 groups) and ticagrelor or colchicine treatment (Tig-2 and Col-2 groups). To further confirm the antithrombotic effects of colchicine, the cross section of tails at the indicated locations (referring to the distance from mouse tail tip) was conducted HE staining. In tail vessels of control group mice, severe thrombosis happened at different locations (Figure 1(d)). For example, at the locations of 3 and 7 cm from the tail tip, the vessel was completely occluded by thrombi. By contrast, colchicine treatment greatly inhibited thrombogenesis. Although a major part of the vessel at the location of 3 cm from the tail tip was occluded by thrombi, almost no thrombus was observed in the 7 cm location. Taken together, these results clearly indicate that colchicine performs both therapeutic and preventive effects on thrombosis in mice.

The endothelium is a monolayer of cells that lines the inside of blood vessels. Endothelial injury exposes collagen that stimulates platelet activation and coagulation. Therefore, the amount of collagen within thrombi is associated with thrombosis [23]. We determined the collagen content within thrombi by Masson staining of tail cross sections at the 3 cm position. Compared with the control group, the intrathrombotic collagen-containing areas were reduced by colchicine (Figure 1(e)).

### 3.2. Colchicine Inhibits Platelet Activation *In Vivo* and Thrombin-Induced Platelet Clot Retraction *In Vitro*

**Figure 3:**
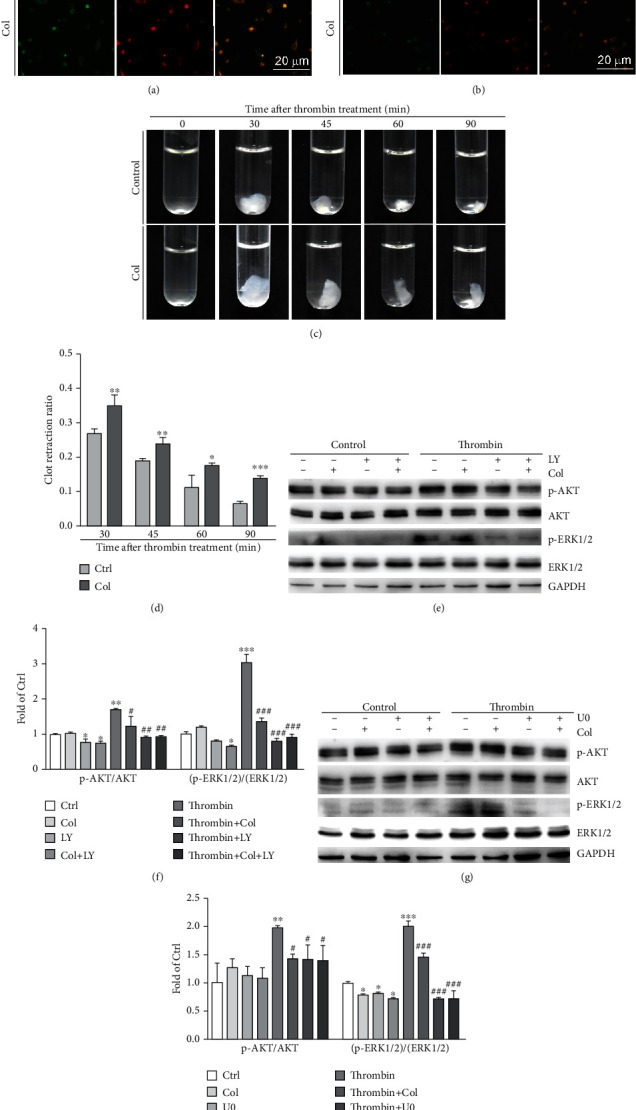
Colchicine inhibits platelet activation *in vivo* and thrombin-induced platelet activation *in vitro*. (a, b) Mice received i.g. administration of colchicine (0.05 mg/day/kg bodyweight) or PBS for 2 days, followed by collection of blood samples. Platelets were collected from mouse blood, then detected expression of CD41 (a) and CD36 (b) by immunofluorescent staining; (c, d) human platelets were treated with CaCl_2_ (1 mM, control group) or CaCl_2_ plus colchicine (100 ng/mL, Col group) at 37°C for 30 min, then added fibrinogen (2 mg/mL) with thoroughly mixed. Thrombin (1 U/mL) was added to the mixture to trigger clot retraction, and clots were photographed at the different time points (c). The spreading area of platelet clot was measured using Photoshop CS6 software (d); (e–h) human platelet suspension was pretreated with colchicine (100 ng/mL), LY294002 (10 *μ*M), U0126 (5 *μ*M), colchicine plus LY294002, or colchicine plus U0126 for 2 h at 37°C, followed by treatment with thrombin (1 U/mL) for 2 min. Expression of p-AKT, AKT, p-ERK1/2, ERK1/2, and GAPDH was determined by Western blot (e, g) with quantitative analysis of band density (f, h). ^∗^*P* < 0.05, ^∗∗^*P* < 0.01, and ^∗∗∗^*P* < 0.001 vs. control; ^#^*P* < 0.05, ^##^*P* < 0.01, and ^###^*P* < 0.001 vs. thrombin-treated group (*n* = 3).

We further investigated the physiological relevance of colchicine on platelet activation. C57BL/6J mice were i.g. administrated with colchicine for two days, followed by determination of the expression of platelet activation markers, CD41 and CD36 in platelets. The results in Figures 3(a) and 3(b) showed that both CD41 and CD36 expression was decreased by colchicine treatment in platelets, suggesting colchicine can attenuate platelet activation *in vivo*. To explore if colchicine can inhibit platelet aggregation, we collected human platelets and treated them with thrombin to trigger platelet clot retraction. As shown in Figures 3(c) and 3(d), human platelet clot reaction was induced by thrombin immediately, while attenuated by colchicine. Results above indicate that colchicine decreases platelet activation in a species-independent manner.

### 3.3. Colchicine Suppresses Human Platelet Activation by Inhibiting the Activation of AKT and ERK1/2

To determine whether colchicine's inhibition on thrombosis is associated with AKT and ERK1/2 pathways, we treated platelets with colchicine, LY294002 (a highly selective inhibitor of AKT), U0126 (an inhibitor of ERK1/2), or their combination in the absence or presence of thrombin. Our results showed that both AKT and ERK1/2 were activated by thrombin. However, colchicine treatment clearly inhibited thrombin-induced p-AKT and p-ERK1/2 levels, while having little effects on total AKT or ERK1/2 (Figures 3(e)–3(h)). Moreover, compared with LY294002 or U0126 alone, the combination of LY294002 or U0126 with colchicine showed no additional benefits on p-AKT or p-ERK1/2 levels. Taken together, these results suggest that colchicine inhibits human platelet activation mainly by inactivating the AKT pathway, which consequently results in inactivation of ERK1/2.

### 3.4. Colchicine Attenuates Human Platelet Activation by Inhibiting ROS Levels

**Figure 4:**
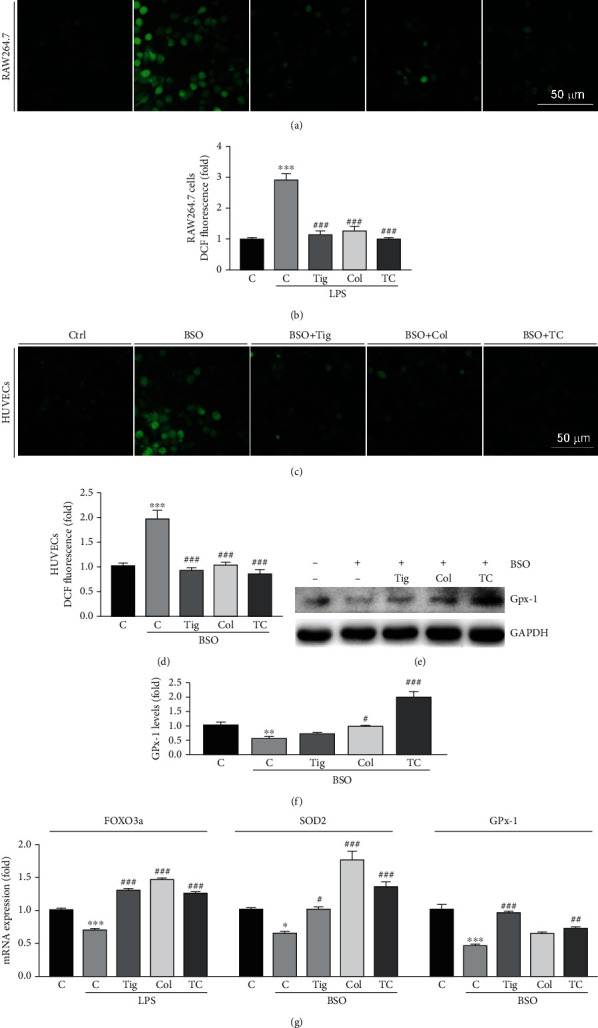
Colchicine inhibits ROS production in HUVECs and RAW264.7 cells by increasing antioxidant enzyme expression. (a–d) RAW264.7 cells were treated with ticagrelor (10 *μ*M), colchicine (100 ng/mL), or ticagrelor plus colchicine in the presence of LPS (500 ng/mL) for 24 h (a, b); HUVECs were treated with ticagrelor (10 mM), colchicine (100 ng/mL), or ticagrelor plus colchicine in the presence of BSO (10 *μ*M) overnight (c, d). After treatment, ROS levels were determined by a Zeiss microscope (a, c) or using a fluorescence microplate reader (b, d); (e–g) HUVECs were treated with ticagrelor (10 mM), colchicine (100 ng/mL), or ticagrelor plus colchicine in the presence of BSO (10 mM) for 24 h. Expression of GPx-1 and GAPDH was determined by Western blot (e) with quantitation of band density (f). Expression of FOXO3a, SOD2, and GPx-1 mRNA was determined by qRT-PCR (g). ^∗^*P* < 0.05, ^∗∗^*P* < 0.01, and ^∗∗∗^*P* < 0.001 vs. control; ^#^*P* < 0.05, ^##^*P* < 0.01, and ^###^*P* < 0.001 vs. LPS or BSO treated group (*n* = 3).

In order to determine whether colchicine or the combination of colchicine and ticagrelor has antioxidative stress effects, we used LPS or BSO to induce ROS levels in RAW264.7 cells or HUVECs, respectively. Our results showed that LPS and BSO enhanced ROS accumulation in macrophages and endotheliocytes, respectively. In contrast, the elevated ROS levels in both RAW264.7 cells and HUVECs were attenuated by colchicine, ticagrelor, or their combination (Figures 4(a)–4(d)). GPx-1 and SOD2 are main antioxidative enzymes to protect the cells from ROS injury. Forkhead box O3 (FOXO3a) is an important transcription factor to regulate antioxidative enzymes. We found that in HUVECs, BSO reduced GPx-1 protein and mRNA expression, as well as SOD2 mRNA levels, which were attenuated by the combination treatment of ticagrelor and colchicine (Figures 4(e)–4(g)). In addition, colchicine, ticagrelor, or their combination could antagonize the BSO-decreased FOXO3a mRNA levels in HUVECs (Figure 4(g)).

**Figure 5:**
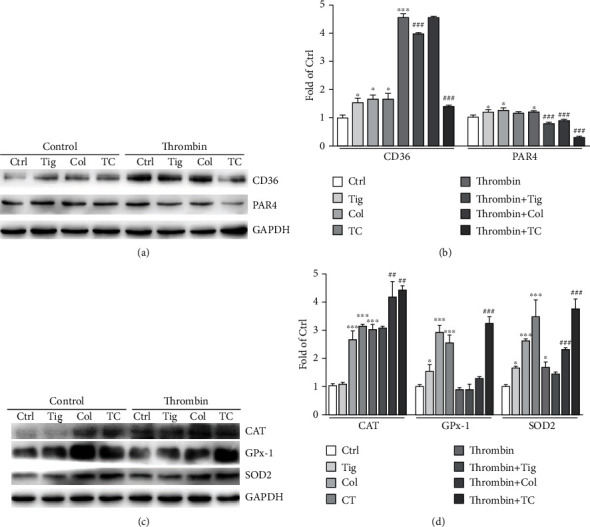
Colchicine attenuates human platelet CD36, PAR4 levels, but enhances antioxidant enzyme expression. (a–d) Human platelets were pretreated with ticagrelor (10 *μ*M), colchicine (100 ng/ml), or ticagrelor plus colchicine for 2 h at 37°C, followed by treatment with thrombin (1 U/mL) for 2 min. Expression of CD36, PAR4, CAT, GPx-1, SOD2, and GAPDH was determined by Western blot (a, c) with quantitation of band density (b, d); ^∗^*P* < 0.05 and ^∗∗∗^*P* < 0.001 vs. control; ^##^*P* < 0.01 and ^###^*P* < 0.001 vs. thrombin-treated group (*n* = 3).

In human platelets, we showed that both ticagrelor and the combination of colchicine and ticagrelor reduced PAR4 and CD36 expression in the presence of thrombin. Moreover, their combination showed better inhibition effects than ticagrelor did (Figures 5(a) and 5(b)). Consistent with the results in HUVECs, colchicine and their combination increased the protein levels of CAT and SOD2 in the absence or presence of thrombin, or GPx-1 in the absence of thrombin (Figures 5(c) and 5(d)). Our results suggest that colchicine reduces oxidative stress by regulating antioxidant enzymes both in HUVECs and platelets.

### 3.5. Colchicine Reduces the Adhesion of Monocytes to HUVECs by Inhibiting the Inflammation Pathway

**Figure 6:**
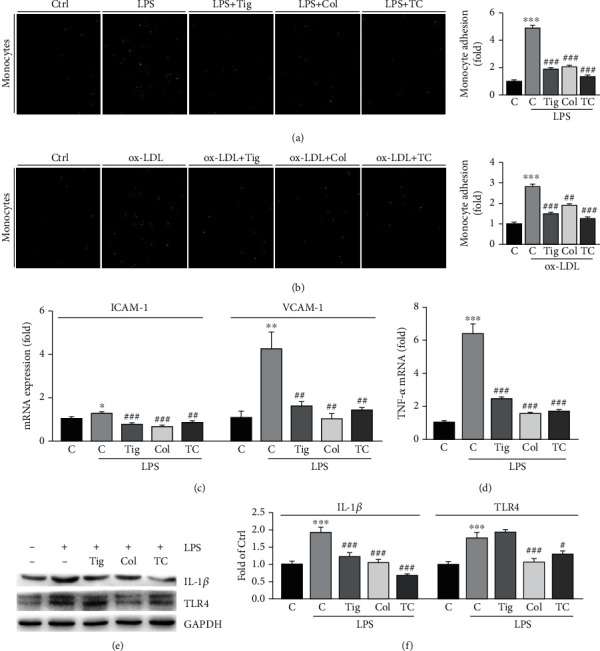
Colchicine inhibits the adhesion of HUVECs with monocytes by regulating the TLR4 pathway. (a, b) HUVECs in 24-well plates were treated with LPS (1 mg/mL), colchicine (100 ng/mL), or LPS plus colchicine for 24 h (a), or ox-LDL (100 *μ*g/mL), colchicine (100 *μ*g/mL), or ox-LDL plus colchicine overnight (b). After treatment, the CFSE-labeled THP-1 monocytes (1 × 10^5^ cells/well) were added to HUVECs and incubated for 1 h at 37°C. After washing away the unadhered THP-1 cells with PBS, labeled cells were photographed by a fluorescence microscope. The number of THP-1 cells in each group was counted and normalized to the number in the control group; (c–f) HUVECs (c) or RAW264.7 cells (d–f) were treated with ticagrelor (10 mM), colchicine (100 ng/mL), or ticagrelor plus colchicine in the presence of LPS (500 ng/mL) for 24 h. Expression of ICAM-1, VCAM-1 (c), and TNF-*α* (d) mRNA was determined by qRT-PCR. Expression of IL-1*β*, TLR4, and GAPDH was determined by Western blot (e) with quantitation of band density (f). ^∗^*P* < 0.05, ^∗∗^*P* < 0.01, and ^∗∗∗^*P* < 0.001 vs. control; ^#^*P* < 0.05, ^##^*P* < 0.01, and ^###^*P* < 0.001 vs. LPS or ox-LDL alone (*n* = 3).

To investigate the inhibitory role of colchicine, ticagrelor, or their combination on monocyte adhesion, we performed cell adhesion assays. As shown in Figures 6(a) and 6(b), both LPS and ox-LDL substantially increased monocyte adhesion to HUVECs. However, colchicine, ticagrelor, and their combination significantly reduced adhesion of monocytes to HUVECs. Meanwhile, we found colchicine, ticagrelor, and their combination blocked LPS-induced expression of adhesion molecules, ICAM-1, and VCAM-1 in HUVECs (Figure 6(c)). Our results also indicated that colchicine, ticagrelor, and their combination blocked LPS-induced inflammatory cytokines, TNF-*α* at mRNA, and IL-1*β* at protein levels in RAW264.7 cells (Figures 6(d)–6(f)). TLR4 is a receptor of LPS, can initiate the innate immune response, and regulate inflammatory cytokine expression. Furthermore, we showed that LPS increased TLR4 protein levels, which were restored by colchicine or the combination of colchicine and ticagrelor (Figures 6(e) and 6(f)). Taken together, we demonstrate that colchicine inhibits monocyte adhesion to HUVECs by inhibiting inflammatory response. In addition, our results also suggest that the combination of colchicine and ticagrelor may have synergic antithrombotic effects.

### 3.6. The Combination of Colchicine and Ticagrelor Works Best on Antithrombosis in Mouse Tissues

**Figure 7:**
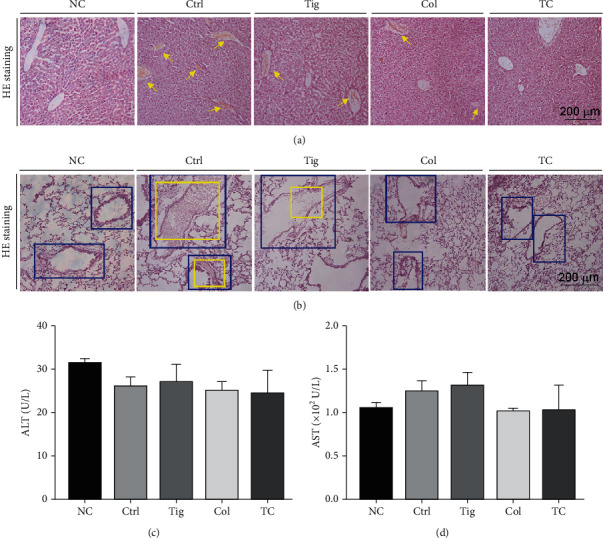
The combination of ticagrelor and colchicine inhibits carrageenan-induced thrombosis in mouse liver and lung tissues. Liver, lung, and serum samples collected from mice used in Figure 2 were conducted the following assays. (a, b) Liver (a) and lung (b) frozen sections were prepared for HE staining. The yellow arrow represents intrahepatic thrombus formation in the liver; blue squares represent the outline of the blood vessel in the lung tissues, and yellow squares represent the intravenous thrombus; (c, d) ALT and AST levels in serum were determined (*n* = 5).

The results above showed that the combination of colchicine and ticagrelor has additional effects on GPx-1, CD36, and PAR4 expression (Figures 4 and 5), compared with the use of either one alone, indicating their combination may perform best on antithrombosis. To further explore the antithrombotic effects of colchicine and ticagrelor combination in mice, we divided mice into 5 groups, with the indicated treatment as shown in Figure 2(a). The results in Figures 2(b) and 2(c) showed that both colchicine, ticagrelor, and their combination reduced the formation rate of tail thrombosis in mice, with the best observed in the combination group. In addition, HE staining of mouse tail cross sections at indicated locations and Masson staining of tail cross sections at the 3 cm position also showed similar results (Figures 2(d) and 2(e)). Carrageenan can induce not only tail thrombosis but also vascular thrombosis in other tissues. Consistent with the results in mouse tail, we found carrageenan caused severe thrombi in liver and lung vessels, with the most significant alleviation in the combination group (Figures 7(a) and 7(b)). Moreover, we found that colchicine, ticagrelor, and their combination caused little side effects, evidenced by little changes of ALT and AST levels in mouse serum (Figures 7(c) and 7(d)). In summary, we showed that the combination of colchicine and ticagrelor works best on antithrombosis in mouse tissues.

## 4. Discussion

In this study, we demonstrate that colchicine can inhibit carrageenan-induced thrombosis in mice and platelet activation (Figures 1–3) and improve thrombin-induced platelet clot retraction *in vitro* (Figures 3(c) and 3(d)). Mechanistically, colchicine reduces AKT and ERK1/2 pathway (Figures 3(e)–3(h)), enhances the properties of antioxidative stress by inducing expression of antioxidant enzymes (Figures 4 and 5), and inhibits the TLR4 pathway to decrease inflammatory cytokines (Figure 6). In addition, we also showed that the combination of colchicine and ticagrelor performs the best protective effects on carrageenan-induced thrombosis, which were evidenced by reduced thrombosis in mouse tail, liver, and lung vessels (Figures 2 and 7). Our study indicates that colchicine may be a new strategy for the treatment of thrombosis. Furthermore, we also suggest that the combination of colchicine and ticagrelor may be a better therapy to consider.

Oxidative stress is associated with many thrombosis-related systemic diseases. It has been shown that ROS are involved in vascular inflammation, with the entire process continuing from the early stages of involvement in immune defense to severe ultimate complications, including thrombosis, tissue hypoxia, and necrosis [24]. In recent years, there have been increasing reports on the relationship between ROS and thrombosis. Some studies have shown that statins exert antithrombotic effects by inhibition of platelet NADPH oxidase-derived ROS formation [25]. Meanwhile, flavonoids can act as antioxidants to reduce ROS levels, thereby inhibiting platelet aggregation and impeding thrombus formation [26]. In this study, we showed that colchicine inhibited LPS or BSO-induced ROS production *in vitro* (Figures 4(a)–4(d)). The expression of antioxidant enzymes can not only prevent the cytotoxic effects caused by ROS but also regulate the oxidation-sensitive signaling pathways in platelets [27]. Our results showed that colchicine alleviates oxidative stress by increasing the expression of antioxidant enzymes, CAT, GPx-1, and SOD2 in HUVECs and platelets (Figures 4 and 5). Thrombosis is associated with endothelial dysfunction. Damaged endothelium releases prostaglandins, adenosine nucleotides, and other intracellular components that enhance platelet aggregation [28]. It has been shown that cigarette smoke can promote thrombosis by causing ROS production and endothelial dysfunction [29]. Our results indicated that colchicine inhibits monocytes' adhesion to HUVECs by reducing the expression of adhesion molecules (Figures 6(a)–6(c)).

In clinic, many critical patients with systemic inflammation usually show coagulation abnormalities [30]. However, thrombosis is not a one-way process. It also significantly promotes inflammation. Thrombin binds to PARs on the surface of macrophages, leukocytes, platelets, and endothelial cells, thereby propagating the thromboinflammatory process. In our study, colchicine was found to reduce the expression of inflammatory factors in RAW264.7 cells by inhibiting TLR4 activation, and the inhibition effect was more pronounced in the combination group (Figures 6(d)–6(f)). The above results indicate that the combination group may perform a greater advantage in inhibiting thrombosis by targeting different pathogenesis.

CD36 is a scavenger receptor that is highly expressed on the surface of platelets [31]. It is involved in a diverse array of physiological and pathological processes *in vivo*, including lipid metabolism, inflammation, and atherogenesis, and promotes thrombosis by activating redox-sensitive signaling molecules [16]. It has been found that dyslipidemia enhanced thrombosis can be corrected in CD36 deficiency mice [32]. Furthermore, increased CD36 expression is correlated with ox-LDL-mediated platelet activation and increased thrombotic risk in human platelets. CD36 can also increase platelet ROS and superoxide production, leading to platelet hyperactivity [33]. CD41 is a platelet surface glycoprotein, involved in platelet adhesion, aggregation, and activation. In our study, the reduction of CD36 and CD41 expression in platelets *in vivo* (Figures 3(a) and 3(b)) and thrombin-induced platelet clot retraction *in vitro* (Figures 3(c) and 3(d)) revealed another important antithrombotic mechanism of colchicine. Oxidative stress not only is associated with inflammatory response and platelet activation but also can upregulate adhesion molecules, such as P-selectins and ICAM-1 [34]. The activated HUVECs produce a number of cell adhesion molecules including VCAM-1 and ICAM-1 to enhance the process of monocytes adhering to HUVECs. Consistent with reduced monocyte adhesion to HUVECs, we observed that colchicine reduced LPS-induced mRNA expression of ICAM-1 and VCAM-1 in HUVECs (Figure 6(c)). These results suggest that colchicine reduces the expression of endothelial cell adhesion molecules by inhibiting inflammatory response, thereby exerting the potential for thrombosis.

However, there are some limitations in this study. We only performed a carrageenan-induced mouse thrombosis model in this research. It is better to use other models, such as FeCl_3_- or electric current stimulation-induced aortic thrombosis mouse model in future studies. In addition, we showed that colchicine ameliorates LPS-activated inflammatory signaling pathway in macrophages and BSO-induced ROS generation in HUVECs, but the key targets on colchicine regulation inflammation and ROS have not been identified, which are necessary for further studies.

## 5. Conclusions

We demonstrate that colchicine can reduce carrageenan-caused thrombosis in mouse tissues and suggest that the combination of colchicine and ticagrelor showed the best antithrombotic properties. *In vitro*, we determine that colchicine reduces human platelet clot retraction and adhesion of monocytes to endothelial cells. Our results indicate colchicine can reduce inflammation and oxidative stress by ameliorating LPS-activated TLR4 signaling in macrophages and BSO-induced antioxidant enzymes in HUVECs. Moreover, our study provides a new antithrombotic strategy.

## Figures and Tables

**Table 1:** Synthesis list of real-time PCR primers.

Gene name	Primer sequence 5′-3′
Homo GAPDH	F GGTGGTCTCCTCTGACTTCAACA R GTTGCTGTAGCCAAATTCGTTGT
Homo FOXO3a	F CAGGCTCAGTGTACCCCATT R AAGCCACCTGAAATCACACC
Homo GPx-1	F TCTCTTCGTTCTTGGCGTTC R CGGGACTACACCCAGATGAA
Homo ICAM-1	F CCACAGTCACCTATGGCAACG R GGCCATACAGGACACGAAGCT
Homo SOD2	F TGACCACCACCATTGAACTT R CGTCACCGAGGAGAAGTACC
Homo VCAM-1	F TGGGAAAAACAGAAAAGAGGTG R GTCTCCAATCTGAGCAGCAA
Mus GAPDH	F ACCCAGAAGACTGTGGATGG R ACACATTGGGGGTAGGAACA
Mus TNF-*α*	F CGTCGTAGCAAACCACCAAG R TTGAAGAGAACCTGGGAGTAGACA

F: forward primer; R: reverse primer.

## Data Availability

The data used to support the findings of this study are included in the article. The authors stated that the data underlying the findings of this manuscript is available to share.
